# Comprehensive analysis of T cell exhaustion related signature for predicting prognosis and immunotherapy response in HNSCC

**DOI:** 10.1007/s12672-024-00921-5

**Published:** 2024-03-02

**Authors:** Wei Zhang, Mei Qu, Chun Yin, Zhiliang Jin, Ya Hu

**Affiliations:** 1 Department of Oncology, Jingzhou Hospital, Yangtze University, Jingzhou, China; 2 Department of Pharmacology, Health Science Center, Yangtze University, 1 Nanhuan Road, Jingzhou, 434023 Hubei China

**Keywords:** Nasopharyngeal carcinoma, T cell exhaustion, Prognosis, Immunotherapy

## Abstract

**Background:**

T cell exhaustion (TEX) signifies a condition of T cell disorder which implicate the therapeutic benefits and prognostic significance in patients with cancer. However, its role in the Head and Neck Squamous Carcinoma (HNSCC) remains incompletely understood.

**Methods:**

The detailed data of HNSCC samples were obtained from The Cancer Genome Atlas (TCGA) database and two Gene Expression Omnibus (GEO) datasets. We computed the expression scores of four TEX-related pathways and detected gene modules closely linked to these pathways, indicating prognostic significance. Following this, regression analyses were performed to select eight genes for the development of a predictive signature. The predictive capacity of this signature was evaluated. Additionally, we examined the relationships between TEX-related signature risk scores and the effectiveness of immunotherapy as well as drug sensitivity.

**Results:**

A novel prognostic model, comprising eight TEX-related genes, was established for patients with HNSCC. The prognostic value was further confirmed using additional GEO datasets: GSE65858 and GSE27020. This signature enables the stratification of patients into high- and low- risk groups, each showing distinct survival outcomes and responsiveness to immunotherapy. The low-risk group demonstrated improved prognosis and enhanced efficacy of immunotherapy. In addition, AZD6482, TAF1, Ribociclib, LGK974, PF4708671 and other drugs showed increased sensitivity in the high-risk group based on drug sensitivity values, offering tailored therapeutic recommendations for individuals with various risks profiles.

**Conclusion:**

In conclusion, we developed a novel T cell exhaustion-associated signature, which holds considerable predictive value for both the prognosis of patients with HNSCC and the effectiveness of tumor immunotherapy.

**Supplementary Information:**

The online version contains supplementary material available at 10.1007/s12672-024-00921-5.

## Introduction

The most common epithelial malignancy of the mucosa in the head and neck region is represented by Head and Neck Squamous Cell Carcinoma (HNSCC). It is characterized by high mobility and mortality rates. The incidence of HNSCC is on the rise and is expected to increase by 30% by 2030 [1]. Its occurrence is closely related to viral infection and carcinogen exposure, especially human papillomavirus (HPV) and Epstein–Barr virus (EBV) [2]. HNSCC is a group of highly heterogeneous cancers, and the early diagnosis is difficult. The majority of HNSCC patients present with locally advanced or metastatic stage, resulting in a dismal prognosis [3]. The conventional therapies for HNSCC include surgery and platinum-based chemoradiotherapy, both of which can lead to severe toxicity [4]. The advent of immunotherapy has dramatically changed the current treatment landscape of HNSCC [5]. However, only a minority of patients may experience benefits from immunotherapy. Hence, there is a pressing need to identify and elucidate the predictive signature and ultimately improve the survival rate for HNSCC.

The involvement of T cell exhaustion in tumor progression and cancer immunotherapy has been a topic of substantial interest. T-cell exhaustion, characterized by effector T-cell dysfunction, was initially recognized by Moskophidis et al. [6]. Despite significant advances in the past decade that have improved our comprehension of the mechanisms, T cell exhaustion remains a broad term [7]. Exhausted T cells exhibit a gradual loss of their ability to generate an immune response and display a memory phenotype, marked by the presence of inhibitory receptors like programmed cell death protein 1 (PD-1) and cytotoxic T-lymphocyte-associated protein 4 (CTLA-4), as well as cytokine secretion disorder [8]. Moreover, exhausted T cells lack the ability to produce effector factors necessary for effectively targeting tumor cells. Secretions of interleukin-2 (IL-2), interferon-γ (IFN-γ), and tumor necrosis factor-α (TNF-α) progressively declines during T-cell exhaustion. For example, IL-2 plays a crucial role as a cytokine essential for T-cell survival and activation, bolstering immune responses against infection and tumors [9]. Additionally, the loss of function in exhaustion progresses hierarchically, marked by the dysfunction of particular pathways, including interleukin (IL)-2, tumor necrosis factor (TNF), interferon-γ (IFN-γ) and cytotoxic potential (CTL) [10]. Typically, T cell exhaustion leads to a decline in molecular and cellular functions, including reduced production of IL-2, diminished cytokine versatility, and impaired proliferative capacity. Subsequently, deficiencies in the production of TNF, IFN-γ, and chemokines occur [11]. The ultimate stage of exhaustion involves the elimination of virus-specific T cells [12].

The treatment landscape for cancer has been transformed by immunotherapies, particularly immune-checkpoint inhibitors (ICIs) and adoptive cell transfer. These advances have sparked significant research and clinical focus on T cell exhaustion [13]. Most patients diagnosed with advanced cancer display a relationship with T cell exhaustion [14]. The immunotherapy can significantly improve the antitumor effects by reversing T cell exhaustion, thereby reinvigorating the impaired immune system [15]. Therefore, the number and functional status of T cells play a pivotal role in determining the effectiveness of immunotherapies. Previous studies have employed TEX-related genes to forecast the prognosis of various human cancers, such as hepatocellular carcinoma [16], esophageal adenocarcinoma [17] and lung cancer [18]. Despite the fact that the quantity, localization, and phenotype of infiltrating T cells have been discovered as biomarkers for HNSCC [19], the prognostic significance of T cell exhaustion-related genes in HNSCC prognosis remains incompletely understood. This study aims to explore the influence of TEX on prognosis and effectiveness of immunotherapy in HNSCC patients.

## Materials and methods

### Date acquisition of information of patients with HNSCC

TCGA Head and Neck Cancer cohort from UCSC Xena (https://ucsc.xena.edu, accessed on 24 February 2023) was obtained to retrieve gene expression data and associated clinical information of 495 patients with HNSCC. Subsequently, external gene expression data and clinical information of 270 patients from GSE65858 and 109 patients from the GSE27020 were downloaded from GEO (https://www.ncbi.nlm.nih.gov/geo/, accessed on 10 March 2023). All the transcriptome expression data matrices were normalized using normalizeBetweenArrays in R for subsequent analysis. The clinicopathological characteristics are provided in Supplementary Table 1. This study did not have to acquire ethical approval because we used information that was already in the public database.

### Activity analysis of TEX-related pathways

We utilized single-sample gene set enrichment analysis (ssGSEA), implemented through the ‘GSVA’ R package [20], to estimate the enrichment score of TEX-related pathways for each patients. Similar to previous studies, TEX-related pathway was represented by interleukin (IL)-2, tumor necrosis factor (TNF), interferon-γ (IFN-γ) and cytotoxic potential (CTL) [21, 22]. The gene sets corresponding to TEX-related pathways were obtained from Molecular Signatures Database (https://www.gsea-msigdb.org/gsea/msigdb, accessed on 24 February 2023). Additionally, we employed the “survminer” R package to depict the survival differences across different enrichment scores, using the median value as the cutoff.

### Weighted gene co-expression network analysis

We conducted Weighted gene co-expression network analysis (WGCNA) using the “WGCNA” R package, a system biology approach aimed at clustering closely related genes into modules and assessing the relevance of these modules to traits of external samples [23]. The soft threshold parameters were determined with a power of 6. Initially, ten modules were identified, and further investigation focused on the RED modules, which exhibited the strongest correlations.

### Functional and gene set enrichment analysis (GSEA)

Gene Ontology (GO) and Kyoto Encyclopedia of Genes and Genomes (KEGG) pathways enrichment analyses were performed utilizing the R package “clusterProfiler” [24].

### Construction and validation of the TEX-related gene signature

Data on patient survival were collected and subjected to further analysis. Specifically, the TCGA cohort was employed to construct the risk model of HNSCC patients. Univariate Cox regression was employed to analyze the impact of these genes on survival outcomes. To prevent omissions, we modified the cut-off P-value to 0.1 [25]. Using R package “glmnet”, the LASSO Cox regression method was then applied to refine the candidate genes and construct the most appropriate signature. The risk score was computed utilizing the subsequent formula: risk score = Coef_1_ × Gene expression_1_ + Coef_2_ × Gene expression_2_ + … Coef_n_ × Gene expression_n_. We divided the patients into two groups using the median value of the risk score. Meanwhile, the Kaplan–Meier (K–M) survival curves were drawn to examine the distinction between the two groups. Multivariate Cox regression analysis was performed to ascertain the independent prognostic relevance of the risk score. To validate the prognostic model’s external applicability, we used additional HNSCC datasets (GSE65858 and GSE27020) for validation.

### Establishment of the nomogram

The univariate and multivariate COX regression analyses were conducted to identify independent indicators of overall survival. Then, a prognostic nomogram was developed through multivariable Cox and stepwise regression analyses, considering age, gender, clinical stage, and risk score. The nomogram plot was showed by “regplot” package. Moreover, the predictive accuracy was estimated using the calibration plots and receiver operating characteristics (ROC) analyses. An area under the curve (AUC) exceeding 0.60 indicated moderate accuracy, while an AUC greater than 0.75 was considered highly accurate for predictions.

### The prediction of immunotherapy response and drug sensitivity

We used the R package “EaSIeR” to acquire the immune response score file and establish the connection between immunotherapy response and risk score [26]. Furthermore, we assessed the cancer immunity cycle status of different risk groups using TIP [27]. Sensitivity scores to different drugs for each sample were calculated using the package “oncoPredict” [28]. Sensitivity score was positivity correlated with the IC50 value. Lower IC50 value indicated higher drug sensitivity and better treatment efficacy.

### Mutation landscape of HNSCC

We gathered somatic mutation profiles from TCGA database in the maf format. Subsequently, utilizing the “maftools” R package, we generated a waterfall diagram to depict the mutation landscape of patients with HNSCC [29].

### Statistical analysis

We performed all statistical analyses using R software (The R Project for Statistical Computing, https://www.r-project.org/). The statistical comparison was conducted using the Wilcoxon test by the “ggpubr” package. The Spearman test was used as a correlation test. To compare overall survival rates between different groups, the Long-rank test was conducted using the “survminer” package. Unless otherwise specified, a p value less than 0.05 was regarded as statistically significant.

## Results

### Identification of prognostic TEX-related pathways

**Fig. 1:**
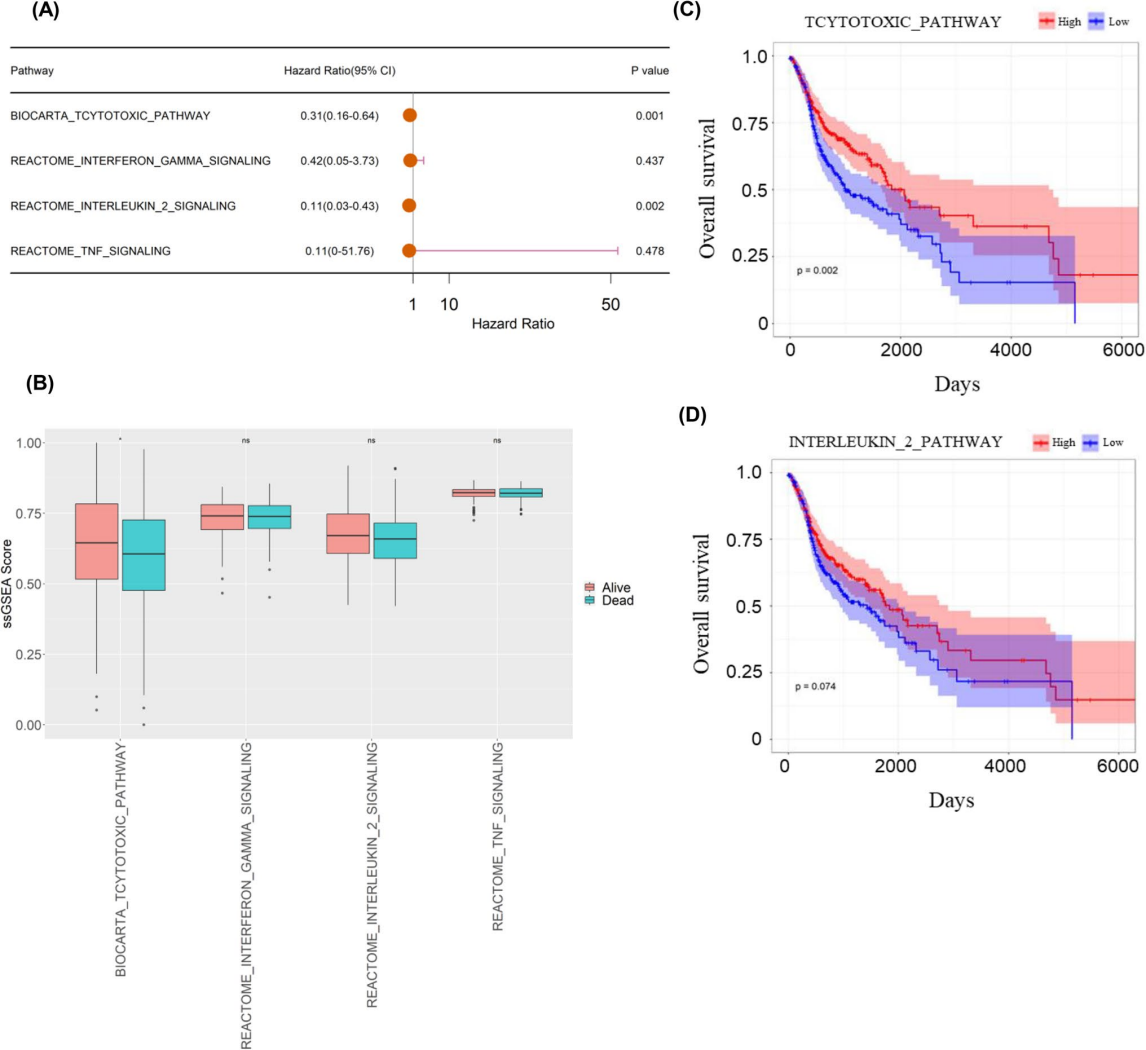
The correlation between TEX-related pathway enrichment scores and prognosis. **A** Univariate cox analysis of TEX-related pathway enrichment scores. **B** The disparity in the enrichment scores of four TEX-related pathways in terms of survival status. **C**, **D** The Kaplan–Meier analysis of enrichment scores in Cytotoxic and IL-2 signaling. (*p < 0.05, **p < 0.01, ***p < 0.001)

Through GSVA analysis, we could investigate variations among subgroups in TEX-related pathways concerning disease prognosis. Firstly, we unraveled the activity of TEX-specific pathways. Next, we aimed to examine the relationship between the pathway enrichment score and prognosis. In the univariate Cox analysis, enrichment scores of Cytotoxic and IL-2 signaling exhibited associations with OS rates of HNSCC (Fig. 1A). In addition, a notable distinction in survival outcomes was observed among the enrichment scores of Cytotoxic signaling (Fig. 1B). In K-M survival analysis, the difference between the groups with high and low scores in Cytotoxic signaling showed significant difference (Fig. 1C). However, the enrichment score of IL-2 signaling was not statistically associated with prognosis (Fig. 1D, P = 0.074). To avoid omissions, these two pathways were selected for subsequent analysis. Additionally, in the genetic variation analysis, mutations were observed in 69 (13.53%) out of the 510 samples, the mutation rate of the above two TEX-related pathways was low, with PTPRC exhibiting the highest mutation frequency (Supplementary Fig. 1).

### Identification of TEX-related gene modules using WGCNA

**Fig. 2:**
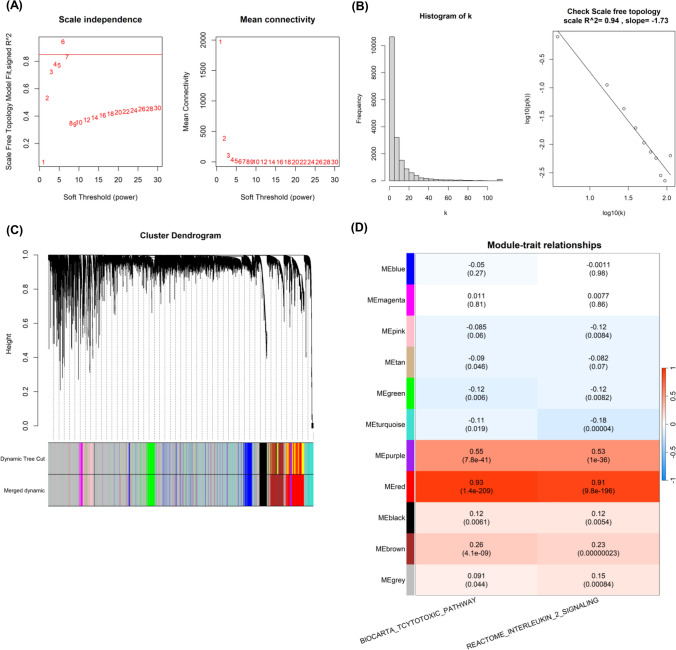
Weighted gene co-expression network analysis. **A** Identification of suitable soft thresholds and validation of a scale-free network. A soft threshold of 6 was chosen. **B** The distribution curve and network connectivity k indicated a satisfactory scale-free network. **C** The cluster dendrogram displayed the gene modules and the merging of modules. **D** Correlation between gene modules and TEX-related pathway

**Fig. 3:**
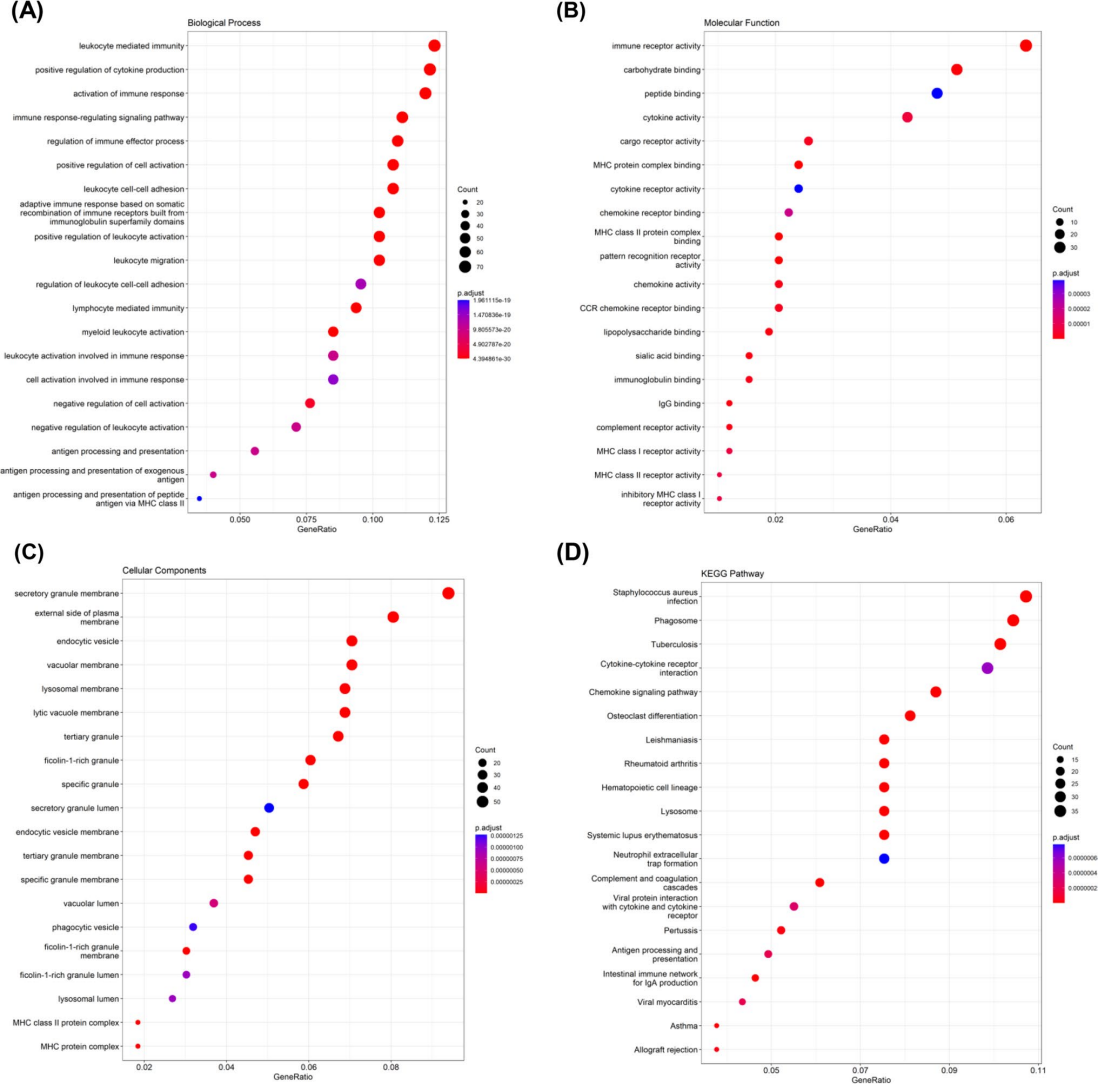
Functional analysis of TEX-related genes. **A**–**C** GO enrichment analysis of 623 TEX-related genes in HNSCC. **D** KEGG pathways analysis of 623 TEX-related genes in HNSCC

RNA-sequencing datasets were employed to identify TEX-associated gene modules through the “WGCNA” package. A scale-free network was constructed using β = 6, which was selected as a suitable soft threshold (Fig. 2A). The dynamic cut tree was created after integrating gene modules that exhibited similarities (Fig. 2B). In total, 10 modules were acquired (Fig. 2C). From the module feature correlation heat map, we noted that the red module exhibited the highest association with the prognostic TEX-related pathways (Fig. 2D), thus we chose the red module for further analysis. There were 623 genes in the red module. In exploring the function of these genes, our findings from the GO functional analysis manifested that the biological processes that exhibited the most significant enrichment among the 623 genes were leukocyte mediated immunity, secretory granule membrane and immune receptor activity (Fig. 3A–C). KEGG pathway analysis revealed notable enrichment in the cytokine-cytokine receptor interaction and chemokine signaling pathway (Fig. 3D). In general, the genes within the red module demonstrated a robust correlation with the immune-related functions in HNSCC.

### Construction of a prognostic TEX-related gene signature for HNSCC patients

**Fig. 4:**
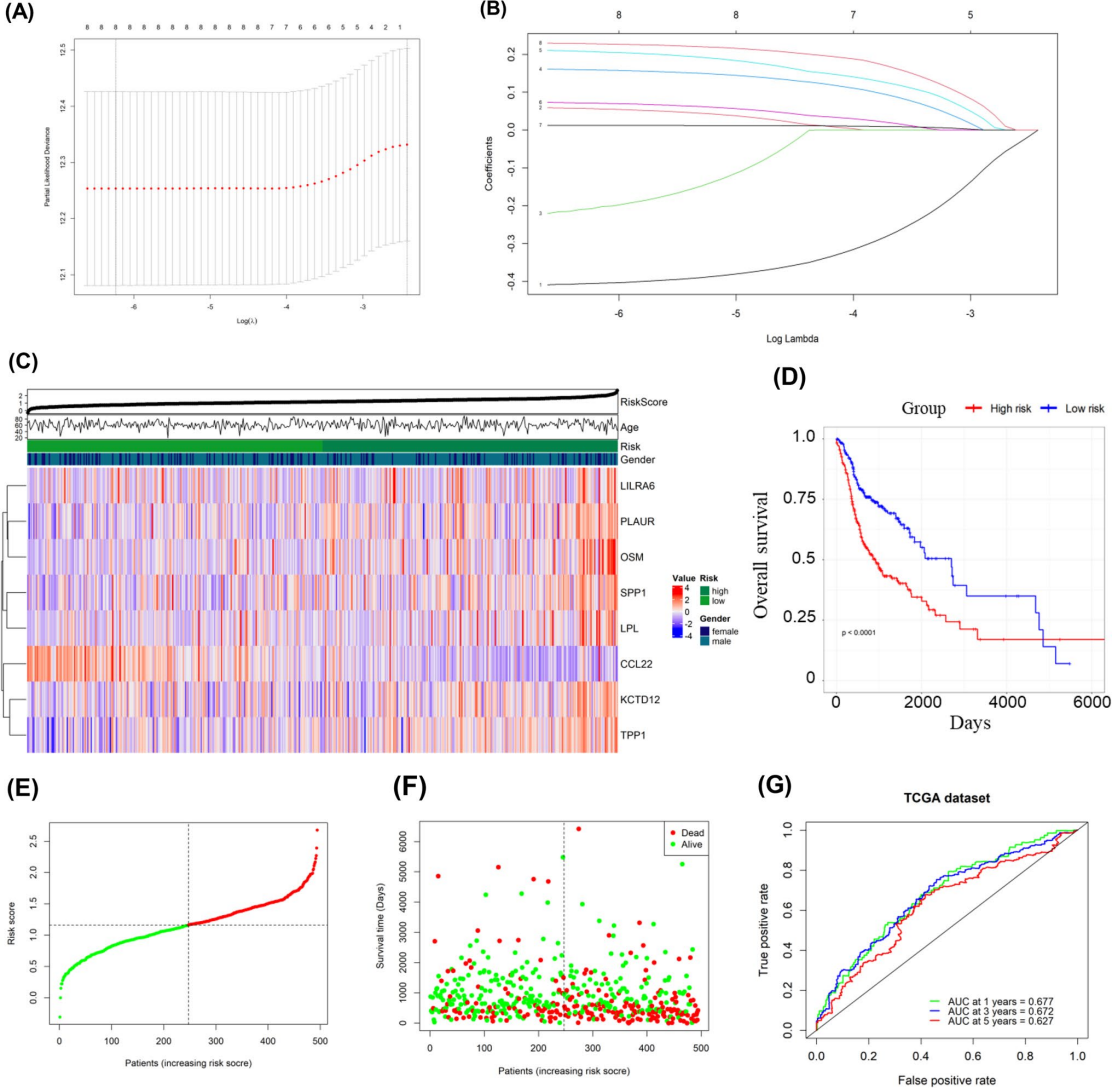
Development and validation of TEX-related risk model for HNSCC patients. **A** Selection of the eight model genes. **B** Cross-validation of the constructed signature. **C** Heatmap of eight model genes and clinical features. **D** Survival curves for high- and low-risk groups decided by the risk score. **E**, **F** Risk score distributions of patients according to the survival status and time. **G** ROC curves in predicting the 1-, 3-, and 5 year survival

**Fig. 5:**
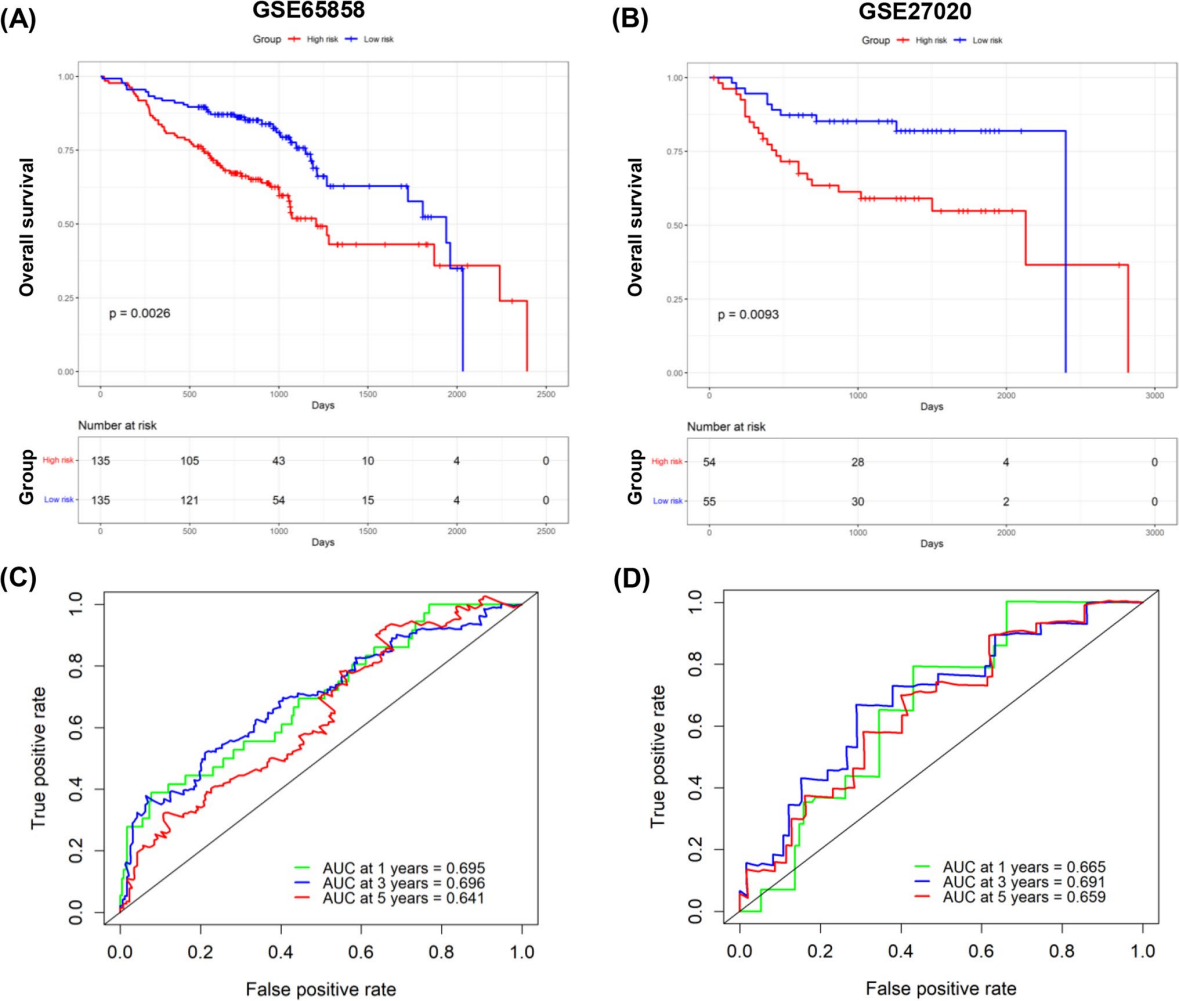
Validation of TEX-related risk model using GEO datasets. **A** K-M survival analysis for high- and low-easier scores groups in GSE65858; **B** K-M survival analysis for high- and low-easier scores groups in GSE27020; **C** ROC curves in predicting the 1-, 3-, and 5 year survival in GSE65858; **D** ROC curves in predicting the 1-, 3-, and 5 year survival in GSE27020

To develop a TEX-related gene signature for HNSCC, the above 623 genes were intersected with the datasets of GSE65858 and GSE27020 to get 416 overlapped genes. Univariate Cox regression analysis screened for survival-related genes. In TCGA-HNSCC, there were 63 genes, and in GSE65858, there were 41 genes that met the cutoff of a P-value less than 0.1. The overlap of these outputs yielded eight genes (CCL22, KCTD12, LILRA6, LPL, OSM, PLAUR, SPP1 and TPP1), which were further used to established a risk model for individual sample stratification. The formula for the risk score was as follow: risk score = (− 0.4059 × CCL22) + (0.0563 × KCTD12) + (-0.2084 × LILRA6) + (0.1588 × LPL) + (0.2073 × OSM) + (0.0712 × PLAUR) + (0.0122 × SPP1) + (0.2281 × TPP1) (Fig. 4A–B). Patients were then classified into two groups. Differential TEX-related genes expression patterns between two groups were illustrated in the heatmap (Fig. 4C). Subsequently, the results of the K-M survival analysis revealed a substantial disparity in OS rates between the two groups, with high-risk HNSCC patients exhibiting unfavorable outcome (P < 0.001; Fig. 4D), indicating the strong predictive capability of our model. Overall survival risk scores distribution, survival time, and status were depicted in Fig. 4E–F. The ROC analysis illustrated the high predictive capability of our model for HNSCC patients, the 1-, 3-, and 5 year AUC was 0.68, 0.67, and 0.63, respectively (Fig. 4G). Additionally, we investigated the prognostic correlation of each gene in the model using K-M analysis (Supplementary Fig. 2). In the validation sets GSE65858 and GSE27020, we observed that samples in high-risk group experienced poor prognosis, and the AUC exhibited an excellent predictive value (Fig. 5). Finally, we showed that risk scores were considerably different in HNSCC patients with various clinical stages and tumor stages (Supplementary Fig. 3).

### Development and evaluation of the nomogram survival model

**Fig. 6:**
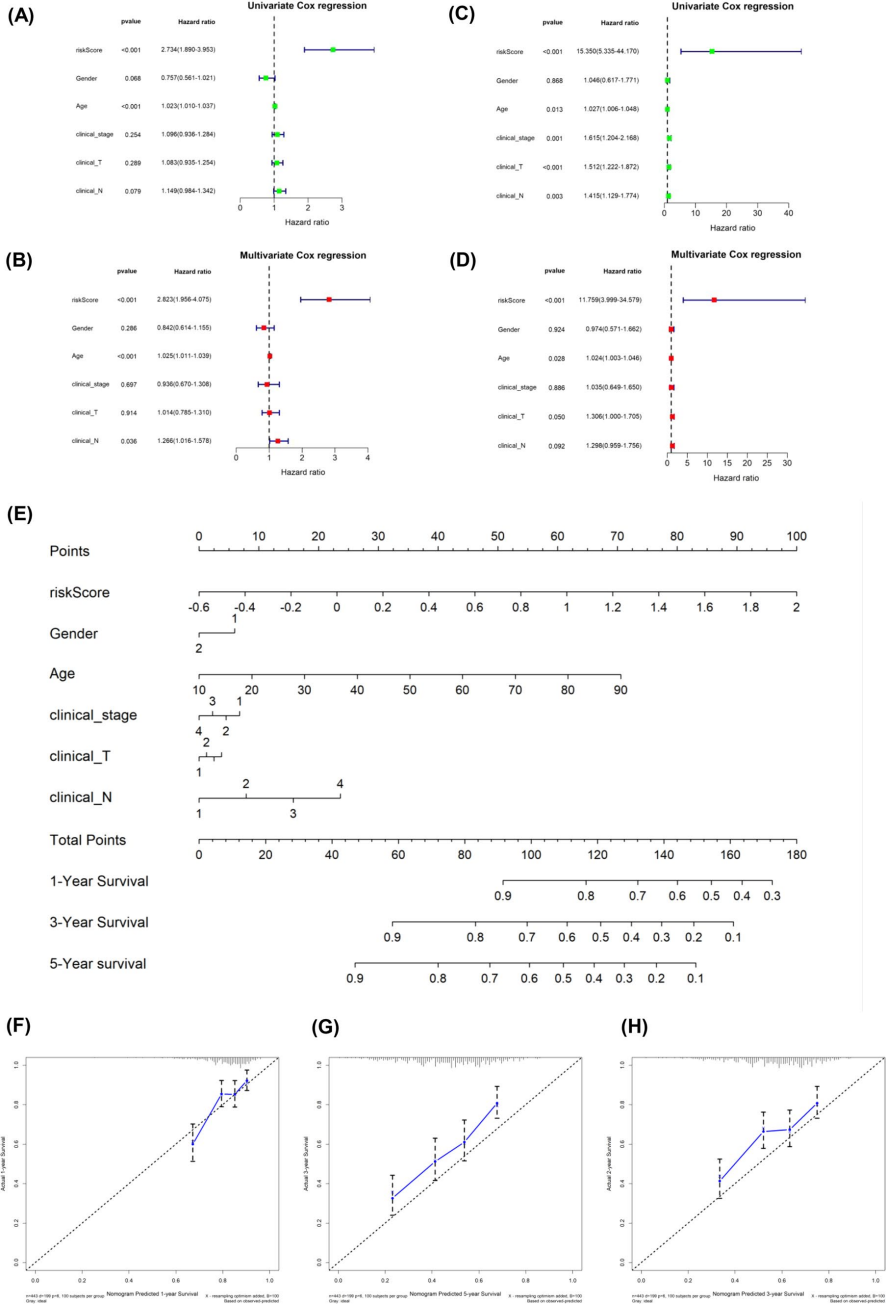
Establishment and calibration of a predictive nomogram. **A**, **B** Univariate and Multivariate analysis were performed for the clinicopathologic characteristics and TEX-related risk scores in TCGA cohort; **C**, **D** Univariate and Multivariate analysis were conducted for the clinicopathologic characteristics and TEX risk scores in GSE65858 dataset; **E** A nomogram was developed for predicting OS in HNSCC patients; **F**–**H** Calibration plots were constructed to assess the performance of nomogram across all samples. **p < 0.01, ***p < 0.001

Afterwards, we examined if TEX-related gene signature could function as an independent prognostic indicator. Univariate Cox regression analysis revealed that TEX-related signature was deemed a hazard factor in comparison to other attributes (HR = 2.73, 95% CI 1.89–3.95, and P < 0.05, Fig. 6A). Moreover, the TEX-related risk score retained its status as an independent prognostic indicator in HNSCC, even after accounting for other confounding variables (HR = 2.82, 95% CI 1.95–4.07, P < 0.05, Fig. 6B). Additionally, the results indicated that TEX-related risk score was likewise an independent predictive indicator in the GSE65858 cohort (Fig. 6C–D). Finally, a nomogram model was developed in the TCGA cohort. This model incorporated sex, age, clinical stage, and risk scores of HNSCC patients (Fig. 6E). As shown in Fig. 6F–H, the precision of this model in forecasting the 1-, 3-, and 5 year survival rates was depicted. These results additionally suggested that this predictive model exhibits superior precision and sensitivity compared to the clinical features.

### Relationship between TEX risk score and response to immunotherapy and drug sensitivity

**Fig. 7:**
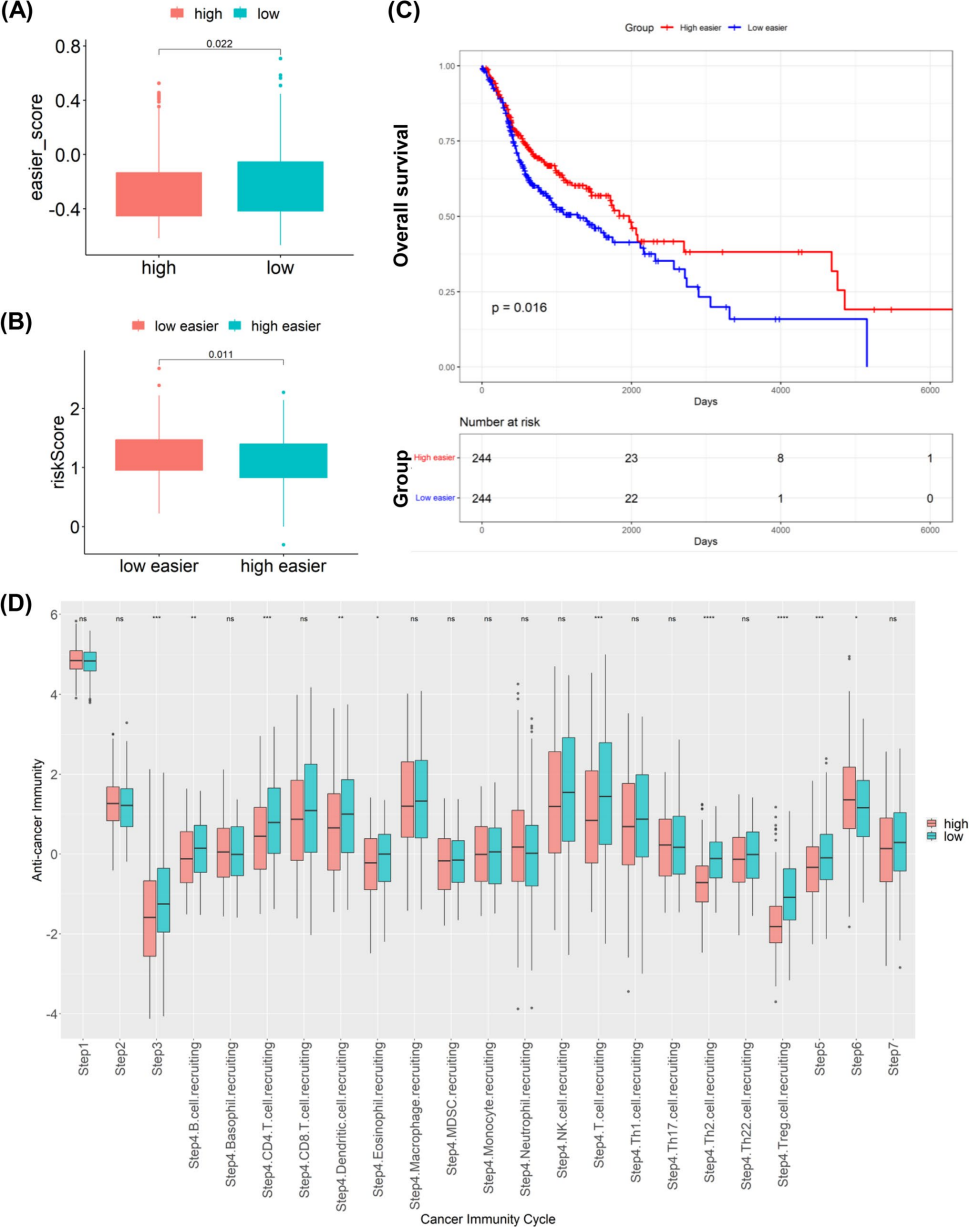
Correlation of risk scores with immunotherapy response and drug sensitivity. **A** Levels of easier scores in high- and low-risk groups, correlation of easier scores with risk scores; **B** Risk scores in high- and low-risk groups, correlation of risk scores with easier scores; **C** K-M survival analysis for high- and low- easier scores groups; **D** The correlation between Cancer immunity cycle and the risk score. *p < 0.05; **p < 0.01; ***p < 0.001; ****p < 0.0001; *ns* no significance

**Fig. 8:**
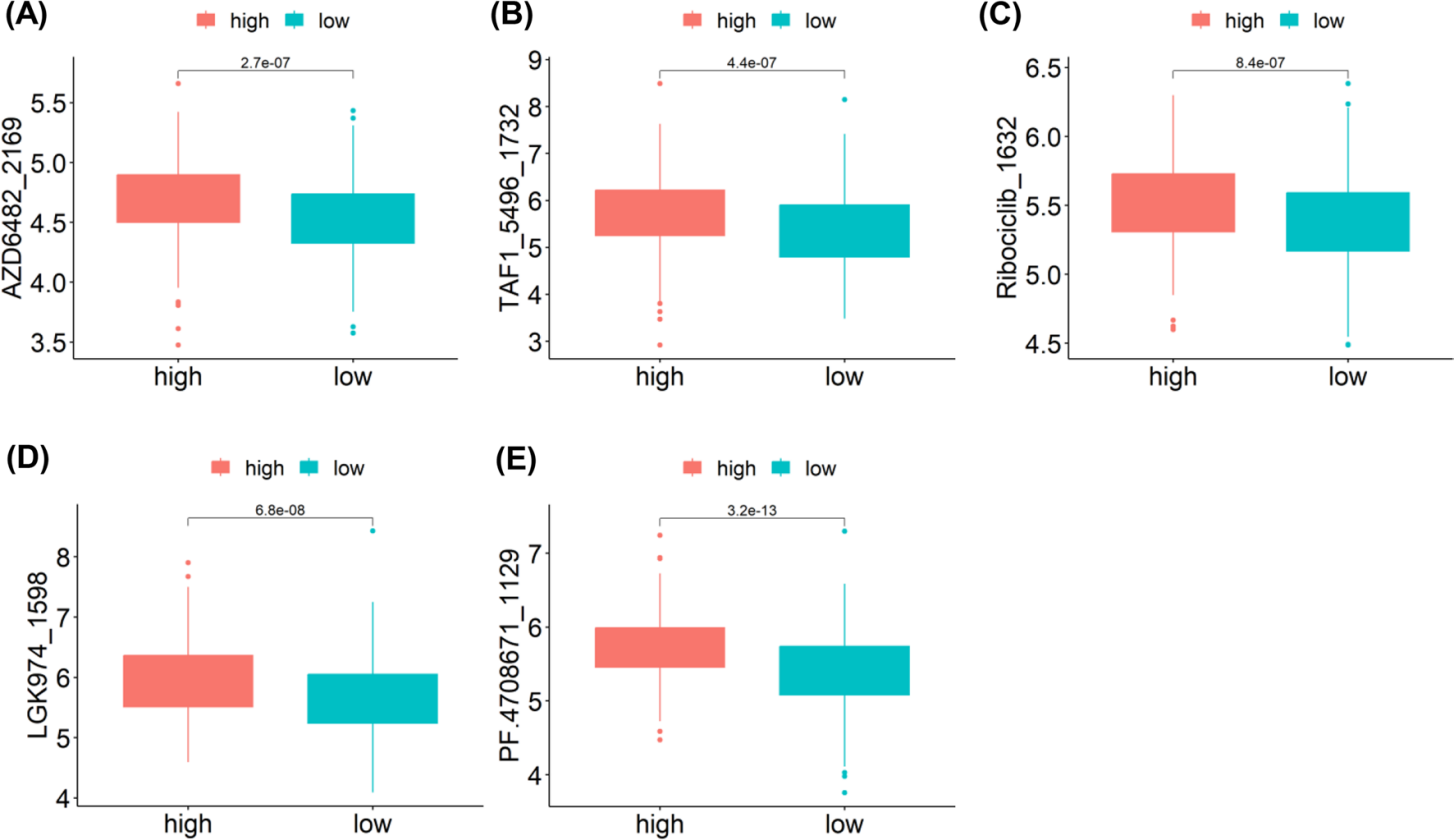
Differences in sensitivity to different drugs in high- and low- risk groups. A AZD6482; B TAF1; C Ribociclib; D LGK974; E PF4708671. *p < 0.05; **p < 0.01; ***p < 0.001; ****p < 0.0001; *ns* no significance

To further explore the relationship between the risk score and immunotherapy response, we evaluated the disparity between the two risk groups based on easier scores, where higher values imply a higher probability of responding positively to immunotherapy. Notably, we noted that easier score was elevated in the low-risk group, and there existed an inverse correlation between easier scores and the risk score (Fig. 7A–B). Moreover, it was evident that the group with low easier scores exhibited a significantly poorer OS rate (Fig. 7C). Therefore, we can conclude that immunotherapy is more probable to be effective in low-risk patients, leading to enhanced outcomes. In addition, leveraging the TCGA-HNSCC cohort and the TIP dataset, subgroup analyses were conducted to evaluate cancer immunity cycle scores in the two groups. There was considerably significant difference in cancer immunity cycle (Fig. 7D), indicating a connection between the tumor immune microenvironment and the risk score, suggesting that TEX may influence the prognosis of HNSCC by modulating the immune cycle state. Together, the prognostic risk score model may be helpful in forecasting patients' response to immunotherapy. Furthermore, to assess the clinical utility of TEX-related score in precise HNSCC treatment, we assessed the effectiveness of commonly prescribed chemotherapeutic drugs in various risk groups. Among those in the high-risk group, AZD6482, TAF1, Ribociclib, LGK974 and PF4708671 appeared to be more sensitive (Fig. 8). These results could potentially offer tailored therapeutic recommendations for individuals with various risks.

### Mutation analysis for each group

Next, we performed a genetic variation analysis. The discrepancy in somatic mutation distribution between the two groups was illustrated. The mutation rates demonstrated similarity between the high-risk (95.06%) and low-risk (92.24%) groups (Supplementary Fig. 4).

## Discussion

T cell exhaustion in HNSCC remains poorly understood, there is a high demand for the identification of more effective biomarkers for immunotherapy and prognosis prediction. In this study, ssGSEA and WGCNA algorithms were employed to filter out the prognostic TEX-related pathways, and then we established an eight-gene signature to forecast the prognosis of HNSCC patients. We illustrated that the risk score model could predict prognosis in HNSCC patients and was successfully validated by independent datasets. The outcomes of the time-dependent ROC analysis demonstrated the outstanding predictive performance. Moreover, a nomogram integrating clinical characteristics and TEX-related risk score was developed, and the findings indicated that the risk prognostic model displayed superior accuracy and sensitivity compared to solely relying on clinical characteristics. The main objective of immune checkpoint blockade is to impede or reverse states of T cell exhaustion, which promoted us to investigate the correlation between TEX risk score and immunotherapy responses. Furthermore, we assessed whether TEX risk score correlated with immunotherapy response, cancer immunity cycle and drug sensitivity. Our results revealed that individuals at high risk exhibited lower easier scores and tend to experience poorer clinical outcomes. This may be explained by the significant difference in cancer immunity cycle between the two groups. Although the specific mechanisms are not fully understood, our results suggest that TEX-related risk score plays a crucial role in forecasting the effectiveness of immunotherapy in HNSCC patients. Besides, we found that HNSCC patients at high risk may benefit from a variety of antineoplastic agents, such as AZD6482, TAF1, Ribociclib, LGK974, PF4708671 and others, it provides different therapeutic recommendations for individuals with different risks.

In a previous study, gene expression profiles of 944 HNSCC patients were examined across four distinct datasets, revealing three separate populations, each characterized by its own unique molecular and immunophenotype, and clinical reactivity. Patients with the highest degree of immune cell infiltration demonstrated the most favorable response to immunotherapy, consequently leading to the most positive prognosis. The results of this study highlighted the significance of the molecular signature, in particular the tumor microenvironment and cytolytic T lymphocytes, in influencing the therapeutic responsiveness and prognosis of HNSCC [30]. Our findings demonstrated that the TEX-related signature independently influenced the prognosis of HNSCC. Furthermore, the model exhibited a good predictive performance for both prognosis and responses to immunotherapy. These results could assist clinicians in making more precise treatment and prognosis assessments for patients with HNSCC.

Most of the eight genes had been reported to be involved in cancer. C–C motif chemokine ligand 22 (CCL22) displays chemotactic activity, attracting T regs to the tumor tissue, and plays a significant role in suppressing T cell immunity [31], it has demonstrated potential as a target in preclinical models [32]. Several studies have indicated that elevated expression of potassium channel tetramerization domain containing 12 (KCTD12) is linked to a positive prognosis in various tumors, including esophageal carcinoma [33] and breast cancer [34]. Lipoprotein lipase (LPL) was identified as a pharmacodynamic biomarker for tumors especially in aggressive breast cancer [35]. In ovarian cancer, Marta et al. discovered that elevated levels of LPL correlated with an unfavorable prognosis [36]. Oncostatin M (OSM) has been identified as significantly linked to tumor progression, potentially serving as a biomarker to predict OS in hepatocellular carcinoma [37]. Elevated levels of urokinase-type plasminogen activator receptor (PLAUR) was detected in bladder urothelial carcinoma, showing a correlation with the abundance of 28 types of tumor-infiltrating lymphocytes [38]. Increased expression of PLAUR indicated an unfavorable prognosis in glioma [39]. Phosphoprotein 1 (SPP1) is a multifunctional phosphorylated glycoprotein secreted by cells. In lung adenocarcinoma, SPP1 has been linked with chemoresistance and unfavorable prognosis [40]. Tripeptidyl peptidase 1 (TPP1) encodes a lysosomal protease capable of cleaving substrate N-terminal tripeptides, it served as an independent predictor for overall survival of hepatocellular carcinoma [41]. Leukocyte immunoglobulin like receptor A6 (LILRA6) has received limited research attention, and its role in tumors remains largely unexplored. Further basic researches are needed to uncover its biological functions. Furthermore, the oncogenic impacts of the prognostic genes in this model, as well as the mechanisms underlying the interaction between prognostic genes and immune dysfunction, remain largely unknown and require further exploration.

While the model we developed exhibits strong performance in predicting the prognosis of patients with HNSCC and aids clinicians in selecting treatment options, it is important to recognize that this study has certain limitations. First, this is a retrospective study, a larger external validation cohort should be employed to confirm the practical application accuracy of the model and its effectiveness in predicting responses to immunotherapy and chemotherapy. Second, the biological function of TEX-related genes in our model has not been investigated in HNSCC in vivo and in vitro, which is essential for further experimental research. However, increasing evidences demonstrated that prognostic signature constructed by multiple genes was more effective and comprehensive than that constructed by single gene in multiple cancers [42]. Our results indicated that TEX-related signature plays a crucial role in predicting immune response and is associated with the prognosis of HNSCC patients.

## Conclusion

In conclusion, we have developed a TEX-related gene signature using the TCGA-HNSCC cohort and confirmed its excellent performance in external cohorts. The risk stratification based on this model accurately predicts the prognosis and immunotherapy response in HNSCC patients, potentially enhancing personalized therapy and improve outcomes. However, further studies are required to validate our findings.

### Supplementary Information

**Additional file 1: Figure S1.** Mutation rate of IL-2 and Cytotoxic signaling in HNSCC.

**Additional file 2: Figure S2.** Kaplan-Meier analysis of each model gene in TCGA-HNSCC.

**Additional file 3: Figure S3.** The association between risk scores and clinicopathological features in TCGA-HNSCC.

**Additional file 4: Figure S4.** The mutation profile in high—and low—risk groups. **A** High-risk group; **B** Low-risk group.

**Additional file 5: Table S1.** The clinicopathological characteristics in HNSCC cases from TCGA and GEO.

## Data Availability

The datasets are available in the TCGA database (https://portal.gdc.cancer.gov/) and GEO database (https://www.ncbi.nlm.nih.gov/geo/).
