# Supplementary figures and images for: Comprehensive analysis of T cell exhaustion related signature for predicting prognosis and immunotherapy response in HNSCC
Source: Discov Oncol. 2024 Mar 2;15:56. doi: 10.1007/s12672-024-00921-5 (PMC10908967; doi:10.1007/s12672-024-00921-5)

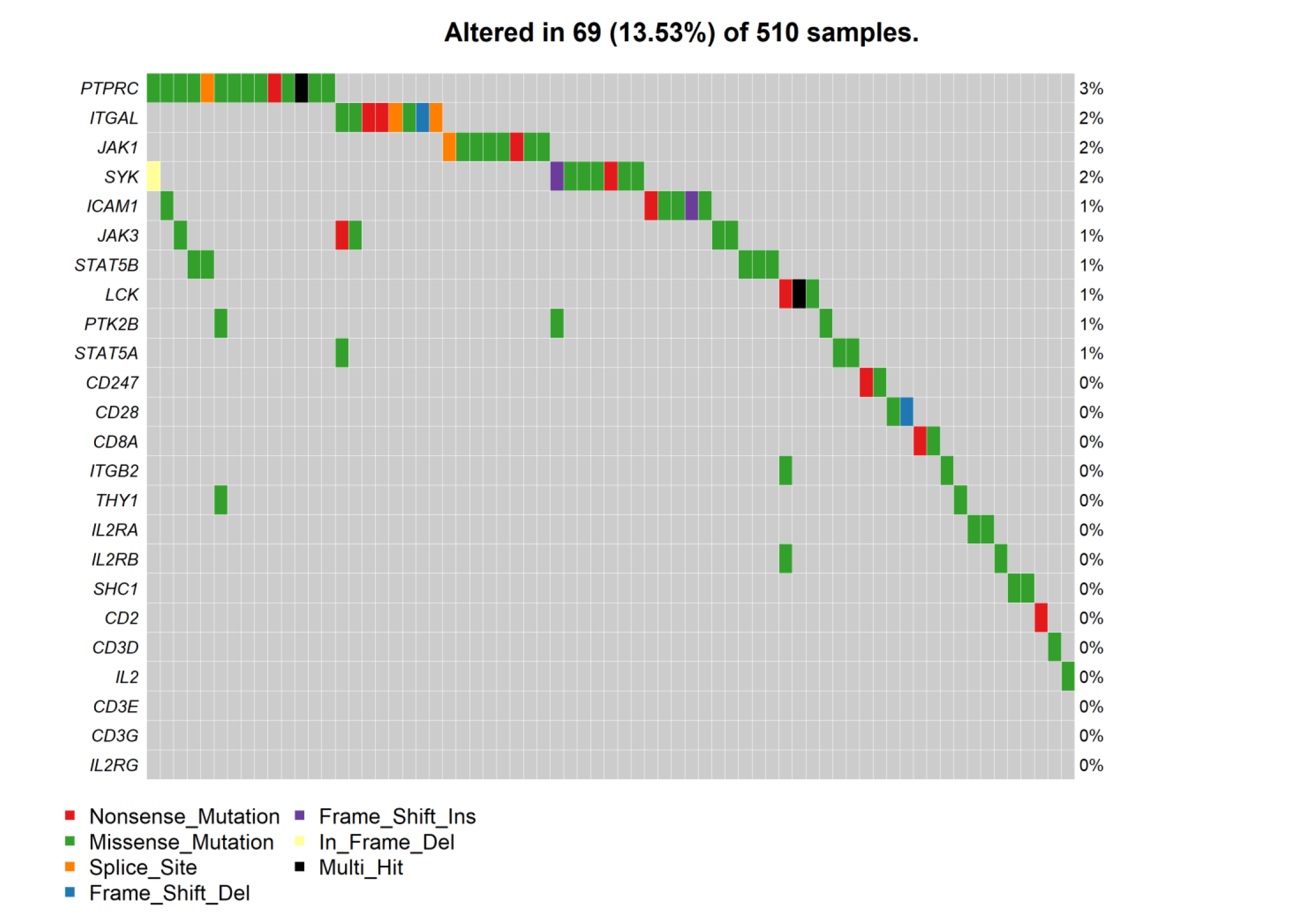

Supplement: Supplementary file 1 — Additional file 1: Figure S1. Mutation rate of IL-2 and Cytotoxic signaling in HNSCC. [file 12672_2024_921_MOESM1_ESM.tif]

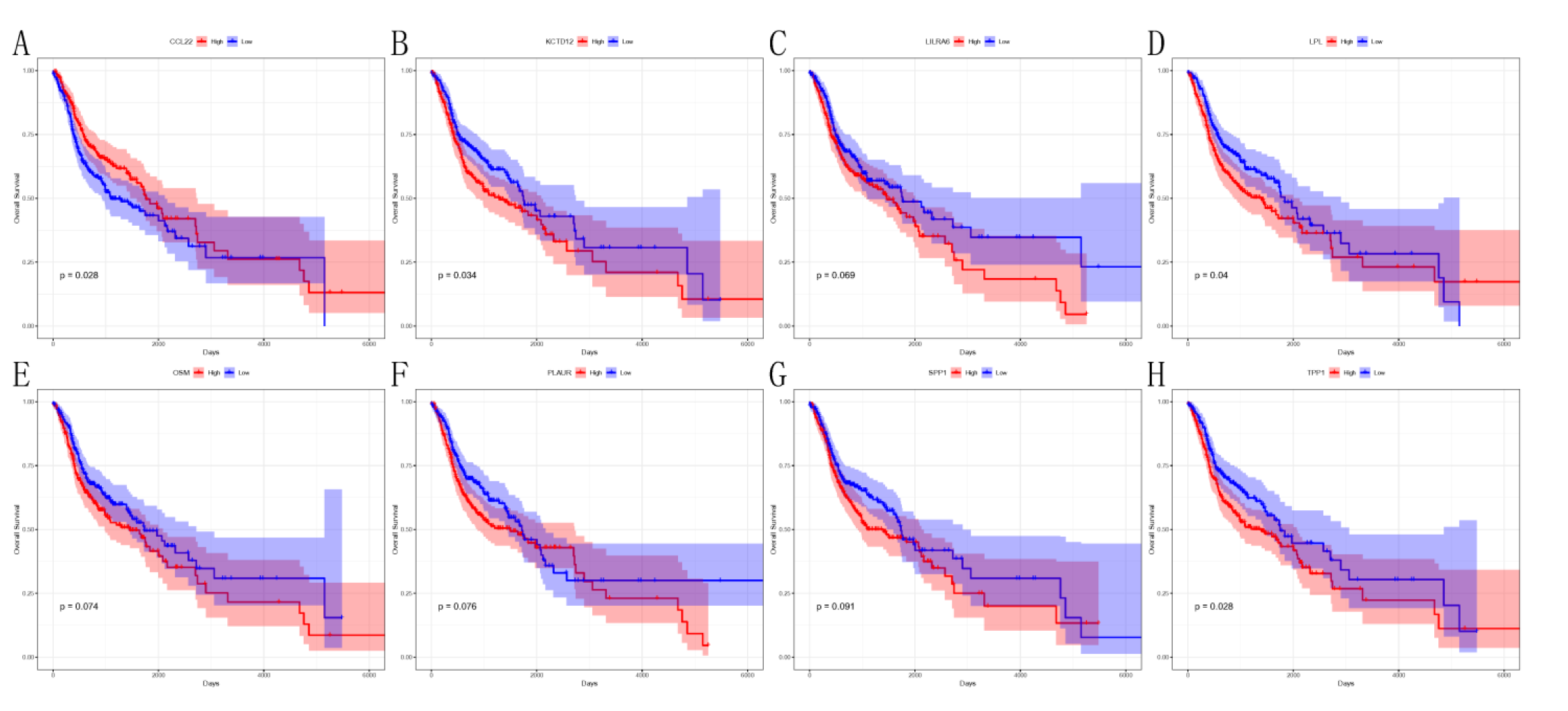

Supplement: Supplementary file 2 — Additional file 2: Figure S2. Kaplan-Meier analysis of each model gene in TCGA-HNSCC. [file 12672_2024_921_MOESM2_ESM.tif]

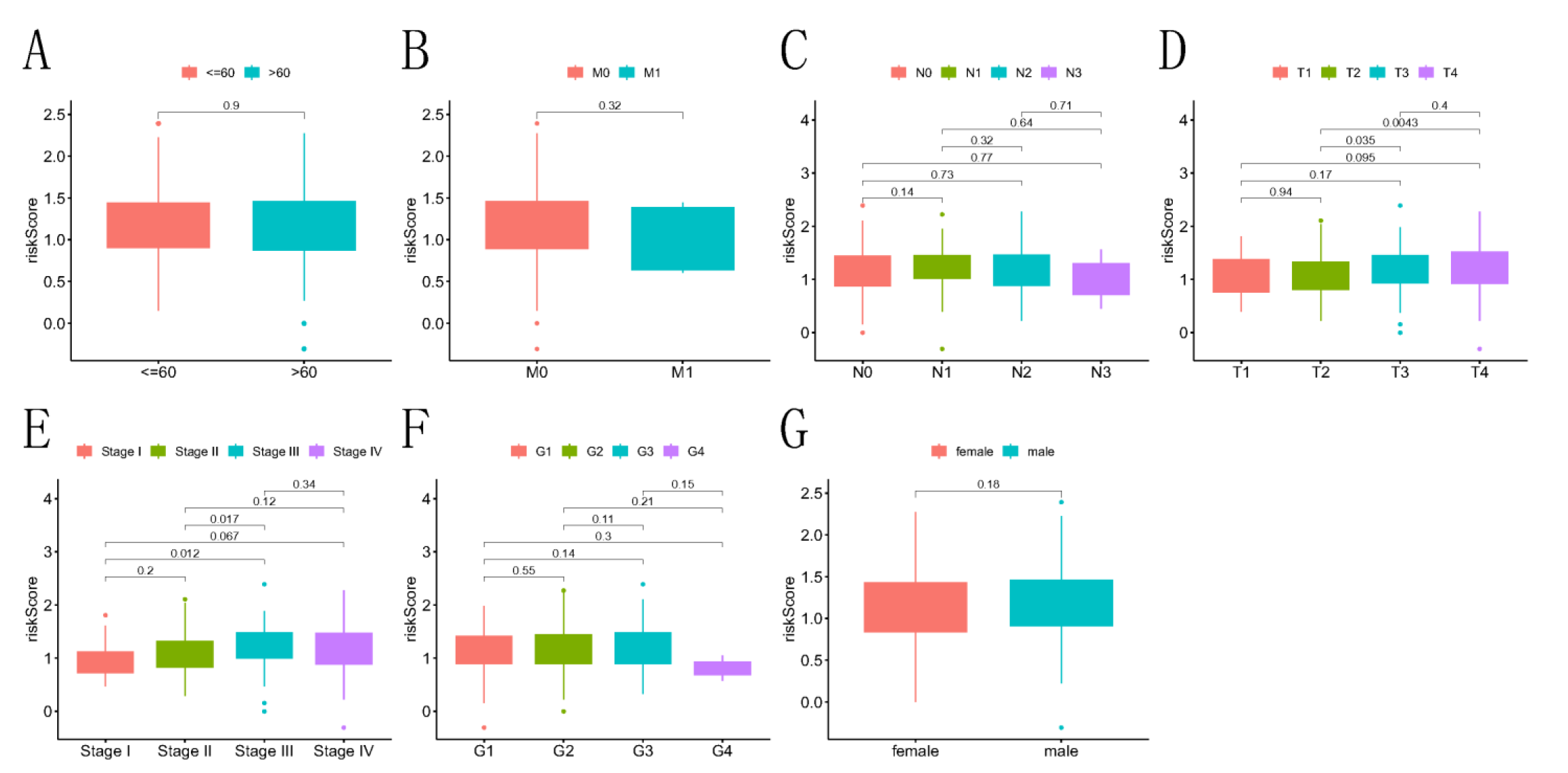

Supplement: Supplementary file 3 — Additional file 3: Figure S3. The association between risk scores and clinicopathological features in TCGA-HNSCC. [file 12672_2024_921_MOESM3_ESM.tif]

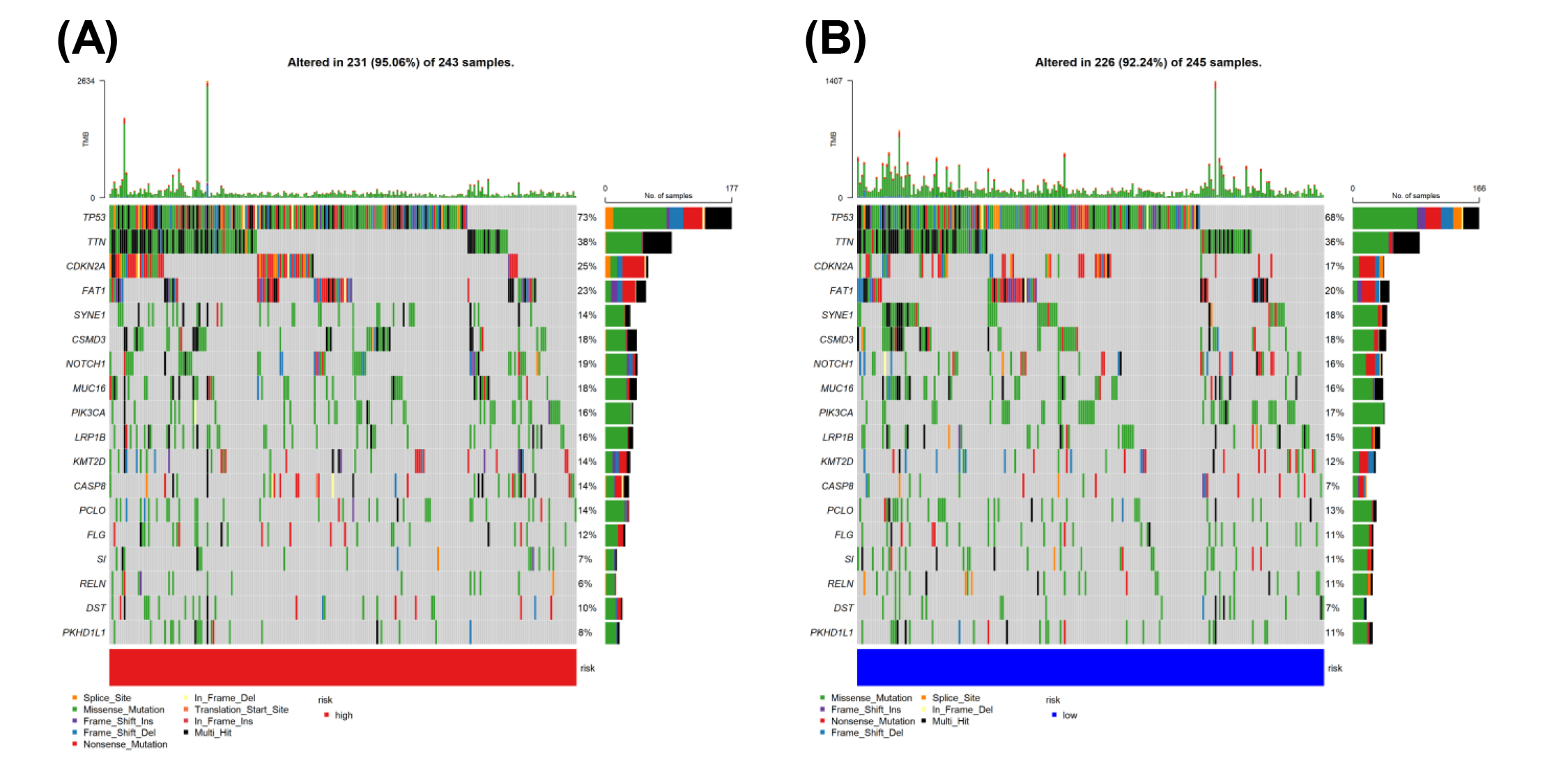

Supplement: Supplementary file 4 — Additional file 4: Figure S4. The mutation profile in high—and low—risk groups. A High-risk group; B Low-risk group. [file 12672_2024_921_MOESM4_ESM.tif]
