# Supplementary material for: Comprehensive analysis of T cell exhaustion related signature for predicting prognosis and immunotherapy response in HNSCC
Source: Discov Oncol. 2024 Mar 2;15:56. doi: 10.1007/s12672-024-00921-5 (PMC10908967; doi:10.1007/s12672-024-00921-5)
Supplement: Supplementary file 5 — Additional file 5: Table S1. The clinicopathological characteristics in HNSCC cases from TCGA and GEO. [file 12672_2024_921_MOESM5_ESM.docx]

**Table 1. The clinicopathological characteristics in HNSCC cases from TCGA and GEO.**

| **Variable** | **TCGA cohort (n = 495)** | | **GSE65858 cohort (n = 270)** | | **GSE27020 cohort (n = 109)** | |
| --- | --- | --- | --- | --- | --- | --- |
| Age | n | % | n | % | n | % |
| <= 60 | 240 | 48.48% | 153 | 56.67% | 43 | 39.45% |
| > 60 | 254 | 51.31% | 117 | 43.33% | 66 | 60.55% |
| Gender |  |  |  |  |  |  |
| male | 363 | 73.33% | 223 | 82.59% |  |  |
| female | 132 | 26.67% | 47 | 17.41% |  |  |
| M |  |  |  |  |  |  |
| M0 | 465 | 93.94% |  |  |  |  |
| M1 | 5 | 1.01% |  |  |  |  |
| MX | 20 | 4.04% |  |  |  |  |
| N |  |  |  |  |  |  |
| N0 | 238 | 48.08% | 94 | 34.81% |  |  |
| N1 | 80 | 16.16% | 32 | 11.85% |  |  |
| N2 | 150 | 30.30% | 132 | 48.89% |  |  |
| N3 | 5 | 1.01% | 12 | 4.44% |  |  |
| NX | 18 | 3.64% |  |  |  |  |
| T |  |  |  |  |  |  |
| T1 | 33 | 6.67% | 35 | 12.96% |  |  |
| T2 | 142 | 28.69% | 80 | 29.63% |  |  |
| T3 | 129 | 26.06% | 58 | 21.48% |  |  |
| T4 | 176 | 35.56% | 97 | 35.93% |  |  |
| TX | 11 | 2.22% |  |  |  |  |
| Grade |  |  |  |  |  |  |
| G1 | 61 | 12.32% |  |  | 42 | 38.53% |
| G2 | 295 | 59.60% |  |  | 49 | 44.95% |
| G3 | 118 | 23.84% |  |  | 16 | 14.68% |
| G4 | 2 | 0.40% |  |  |  |  |
| GX | 16 | 3.23% |  |  |  |  |
| Stage |  |  |  |  |  |  |
| Stage I | 19 | 3.84% | 18 | 6.67% |  |  |
| Stage II | 94 | 18.99% | 37 | 13.70% |  |  |
| Stage III | 102 | 20.61% | 37 | 13.70% |  |  |
| Stage IV | 266 | 53.74% | 178 | 65.93% |  |  |
